# Hematopoietic stem/progenitor cells are less prone to undergo apoptosis than lymphocytes despite similar DNA damage response

**DOI:** 10.18632/oncotarget.16455

**Published:** 2017-03-22

**Authors:** Matus Durdik, Pavol Kosik, Jana Kruzliakova, Lukas Jakl, Eva Markova, Igor Belyaev

**Affiliations:** ^1^ Laboratory of Radiobiology, Cancer Research Institute, Biomedical Center, Slovak Academy of Sciences, Bratislava, Slovakia

**Keywords:** imaging flow cytometry, imagestream, flow cytometry, apoptosis, γH2AX

## Abstract

Hematopoietic stem/progenitor CD34+ cells (HSPC) give rise to all types of blood cells and represent a key cellular target for origination of leukemia. Apoptosis and repair of DNA double strand breaks (DSB) are vital processes in leukemogenesis. High doses of ionizing radiation are the best known agent that induces leukemia, but less is known about the leukemogenic potential of low doses. While umbilical cord blood (UCB) serves as a valuable source of the HSPC for both research and clinics, the data on DNA damage response and apoptosis in UCB HSPC are very limited. We have studied apoptosis and DSB in the UCB-derived CD34+HSPC and CD34- lymphocytes at different time points post-irradiation with low and therapeutic doses of γ-rays. DSB were enumerated with γH2AX foci using imaging flow cytometry. Different stages of apoptosis were analyzed using Annexin/7-AAD assay and γH2AX pan-staining by flow cytometry and imaging flow cytometry, respectively. Our results have consistently shown significantly higher resistance of CD34+ stem/progenitor cells to endogenous and radiation induced apoptosis as compared to CD34- lymphocytes. At the same time, no statistically significant difference was found in DSB repair between HSPC and lymphocytes as enumerated by the γH2AX foci. To conclude, we show for the first time that hematopoietic stem/progenitor cells are less prone to undergo apoptosis than lymphocytes what may be accounted for higher expression of anti-apoptotic proteins in CD34+ cells but was unlikely dealt with DSB repair.

## INTRODUCTION

Human hematopoiesis is initiated from the hematopoietic stem/progenitor cells (HSPC), by their division into more differentiated subtypes. HSPC are known by their multipotency and ability to self-renew. They are characterized by the expression of CD34 and other surface markers in dependence on the differentiation stage [1]. HSPC are responsible for origination of childhood leukemia, which occurs due to accumulation of mutations in hematopoietic stem cells [2]. Unrepaired or misrepaired DNA double strand breaks (DSB) are vital events in formation of various mutations including preleukemic fusion genes. These fusion genes were detected in umbilical cord blood (UCB) [3], which is one of the most available sources of HSPC used both in science and medicine. UCB derived CD34+ cells have been already used for the autologous transplantation in the treatment of childhood leukemia [4]. Phosphorylated form of histone H2AX (γH2AX) is a generally accepted molecular marker for detecting DSB by enumeration of so-called DNA repair foci [5]. Ionizing radiation (IR) at high doses is one of the best known etiological agents that induce leukemia [6]. The radiosensitivity of different HSPC's subpopulations to high doses has been studied in mice [7] and human [8–11]. While irradiation at high doses is relatively rare, HSPC are often exposed to low doses of IR at the medical examinations such as computer tomography and mammography, at airport security controls and at locations with increased radioactive background. Adverse effects of low doses and their leukemogenic potential are not fully understood, but some links between exposure to low doses and leukemia have been described [12, 13]. Several studies reported significant induction of DSB in human differentiated cells at the low doses comparable with those used at the medical examinations [11, 14–16]. However sensitivity of CD34+ HSPC to low-dose radiation induced apoptosis and DNA damage is not fully assessed.

There are significant differences in expression of crucial regulators of apoptotic pathways between CD34+ HSPC and CD34- UCB cells, which may suggest higher resistance of CD34+ cells to apoptosis [17–19]. The aim of this study was to compare the response of UCB hematopoietic stem/progenitor CD34+ cells and lymphocytes to irradiation with γ-rays with low and therapeutic doses.

## RESULTS

### Apoptosis in CD34+/− UCB cells

**Figure 1:**
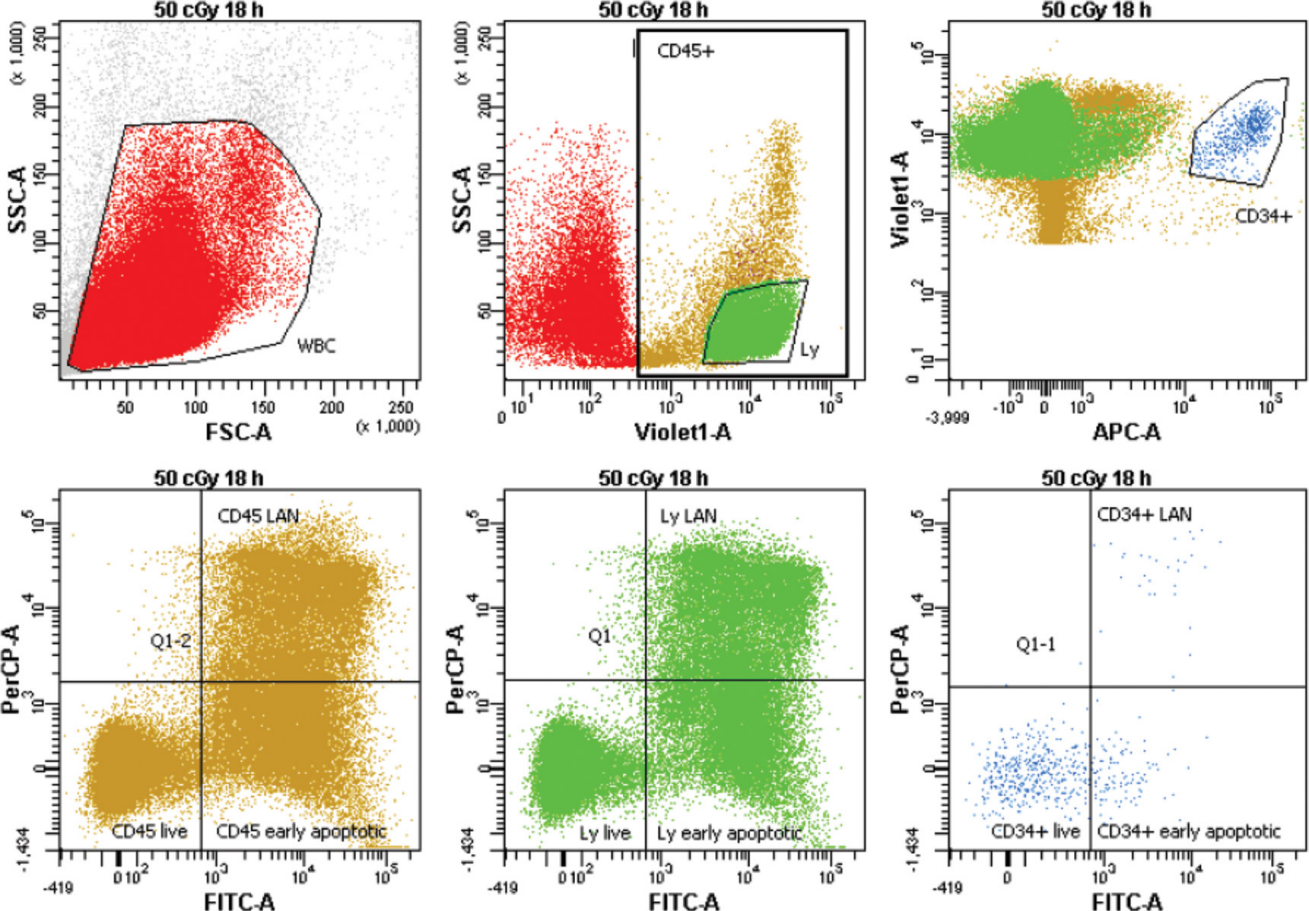
Gating strategy for analysis of apoptosis/necrosis MNC were gated on FSC vs. SSC. Next, CD45 positive, lymphocytes (Ly) with high expression of CD45 and low SSC, and CD34+ cells with high expression of CD34 were gated. Finally, all subpopulations were analyzed on the Annexin/7-AAD scatter for live, early apoptotic, and late apoptotic/necrotic (LAN) cells.

**Figure 2:**
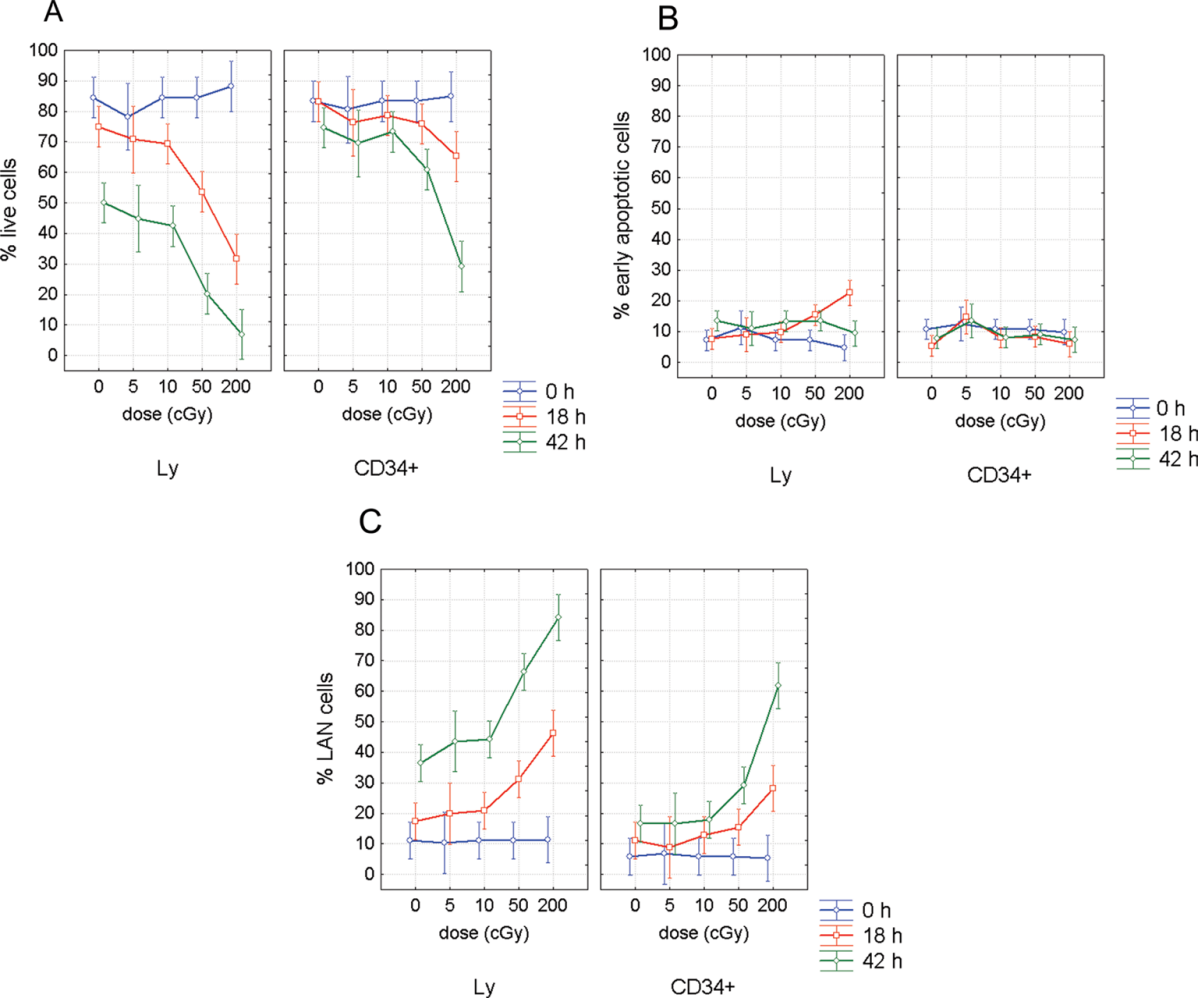
Apoptosis in CD34+/− UCB cells Figure shows percentage of live (**A**), early apoptotic (**B**), and LAN cells (**C**), in lymphocytes (Ly) and CD34+ cells at different time points post-irradiation with γ-rays at doses of 0, 5, 10, 50, and 200 cGy. Mean value and 95% confidence interval is shown from 11 experiments for 0, 10 and 50 cGy, from 7 experiments for 2 Gy, and from 4 experiments for 5 cGy.

We analyzed fractions of live, early apoptotic and late apoptotic/necrotic (LAN) cells using Annexin/7-AAD assay by flow cytometry (Figure 1). This analysis revealed much higher radioresistance of the CD34+ HSPC (Figure 2). CD marker, time after irradiation and dose were significant factors affecting rate of apoptosis (ANOVA, *p <* 0.0000001). Interestingly, both CD marker and incubation time were also significant factors for apoptosis in unirradiated samples (ANOVA, *p <* 0.00002). The role of CD marker was further evaluated by pairwise comparison of data between CD34- and CD34+ cells using two tailed *t-test*. Percentage of live cells was significantly higher in CD34+ population 18 h after irradiation with 50 and 200 cGy (*p* = 0.001 and 0.005, respectively) (Figure 2A). This difference in radioresistance between CD34+ HSPC and lymphocytes became even stronger with time after irradiation. At the later stage of apoptosis, 42 h post irradiation, significantly higher survival of CD34+ cells was observed at all irradiation doses of 5, 10, 50, and 200 cGy (*p* = 0.0001, 0.0003, 0.000001, and 0.002, respectively) and even in the unirradiated control cells (*p* = 0.0004). Analysis of the early apoptotic cells (Annexin-V positive/ 7-AAD negative, Figure 2B) has shown significantly lower radiosensitivity of CD34+ cells at the doses 50 and 200 cGy (*p* = 0.02 and 0.001 respectively) 18 h after irradiation. Analysis of LAN cells (Annexin-V and 7-AAD positive) has confirmed the data obtained by analyzing live cells: (i) significantly higher radioresistance of CD34+ cells in comparison to lymphocytes was detected already 18 h post-irradiation with 50 cGy (*p* = 0.005); (ii) CD34+ were more resistant to late apoptosis/necrosis at all doses 0, 5, 10, 50, and 200 cGy (*p* = 0.0007, 0.002, 0.00005, 0.00001, and 0.01, respectively) as detected 42 h after irradiation. There was also significantly higher endogenous late apoptosis/necrosis in the lymphocyte population of the control samples at the beginning of experiments (*p* = 0.005) (Figure 2C). Given all obtained data, we conclude that UCB hematopoietic stem/progenitor CD34+ cells are more resistant to both radiation-induced and endogenous apoptosis in comparison to CD34- lymphocytes.

### DNA repair foci in CD34+/− UCB cells

**Figure 3:**
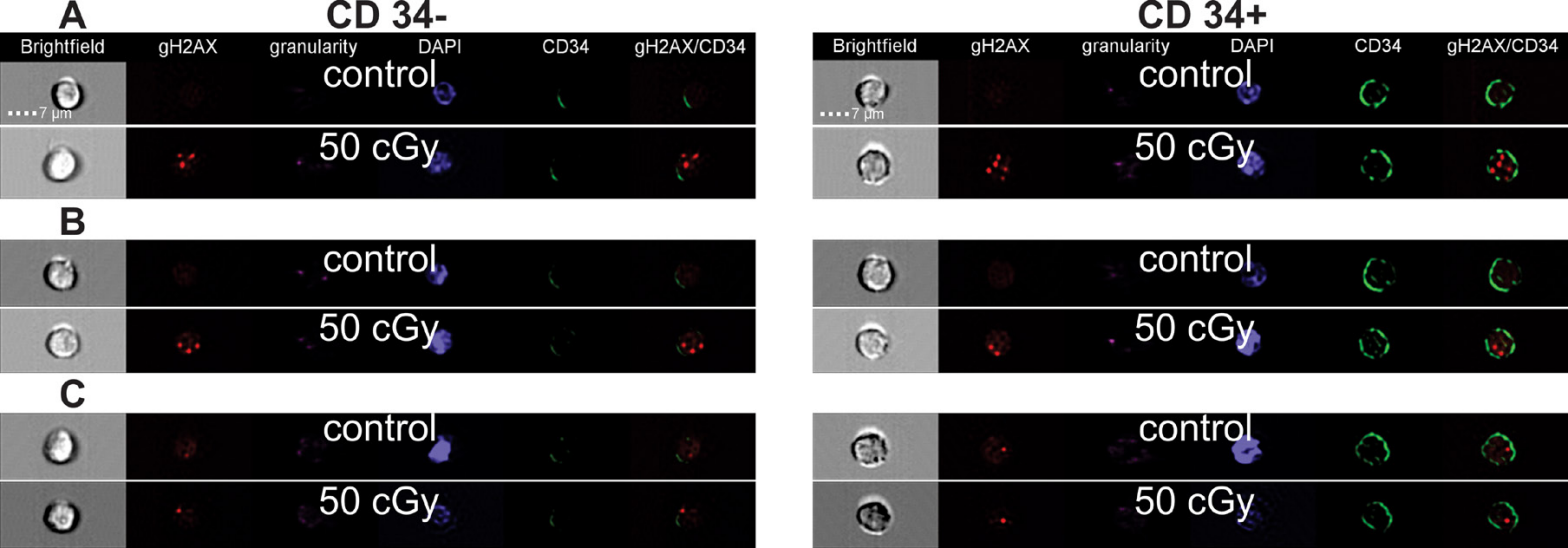
Representative images of cells Representative images of CD34+ HSPC (right column) and CD34- Ly (left column) acquired by the imaging flow cytometry. The irradiated (50 cGy) and control cells at 30 min (**A**), 2 h (**B**), and 18 h (**C**) post-irradiation are shown.

**Figure 4:**
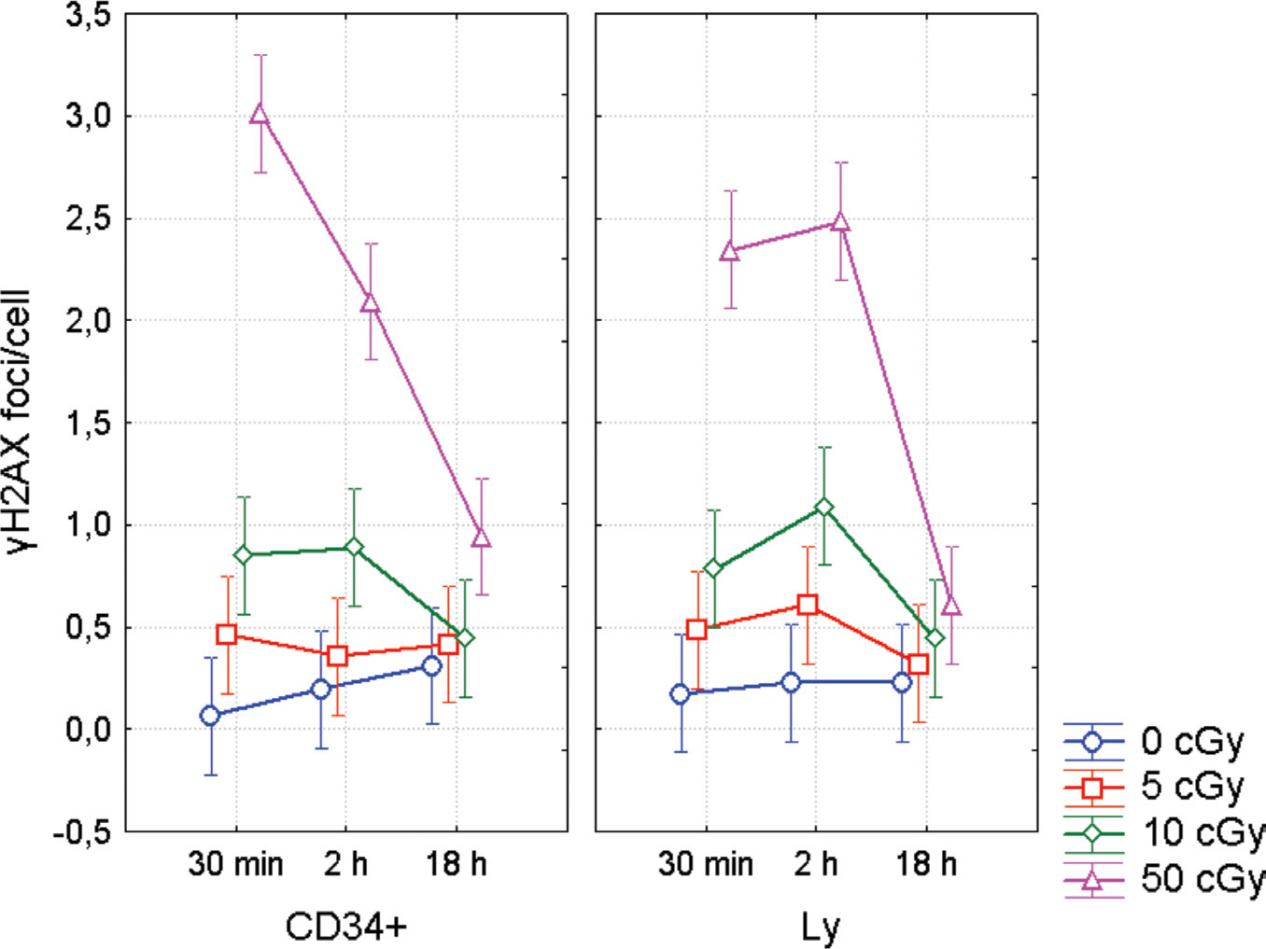
DNA repair foci in CD34+/− UCB cells γH2AX foci enumerated by the imaging flow cytometry. UCB MNC were irradiated with γ-rays (0, 5, 10, and 50 cGy) and then lymphocytes (Ly) and CD34+ HSPC were analyzed at different time points (30 min, 2 and 18 h) after irradiation. The mean value from 4 experiments along with 95% confidence interval is shown in each data point.

Enumeration of γH2AX foci in CD34+ and CD34- populations was performed by the imaging flow cytometry (Figure 3A–3C). In CD34+ cells, we observed maximum level of γH2AX 30 min post irradiation, while γH2AX foci reached their maximum 2 h post-irradiation in CD34- cells (Figure 4). However, statistical analysis of all γH2AX data has not shown any significant difference in DNA damage response between CD34+ HSPC and CD34- lymphocytes (ANOVA, *p* = 0.73). Still there was a trend to lower number of endogenous foci in CD34+ cells (*t-test*, *p* = 0.04).

### γH2AX pan-staining in CD34+/− UCB cells

**Figure 5:**
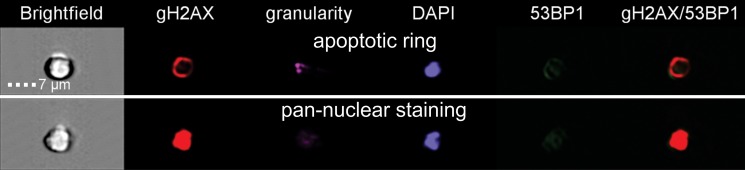
γH2AX pan-staining types Representative images of two types of γH2AX pan-staining in UCB cells are shown. The first one was staining only on the circuit of cells, so called apoptotic ring, and the second one was homogeneous pan-nuclear staining of the whole nucleus.

**Figure 6:**
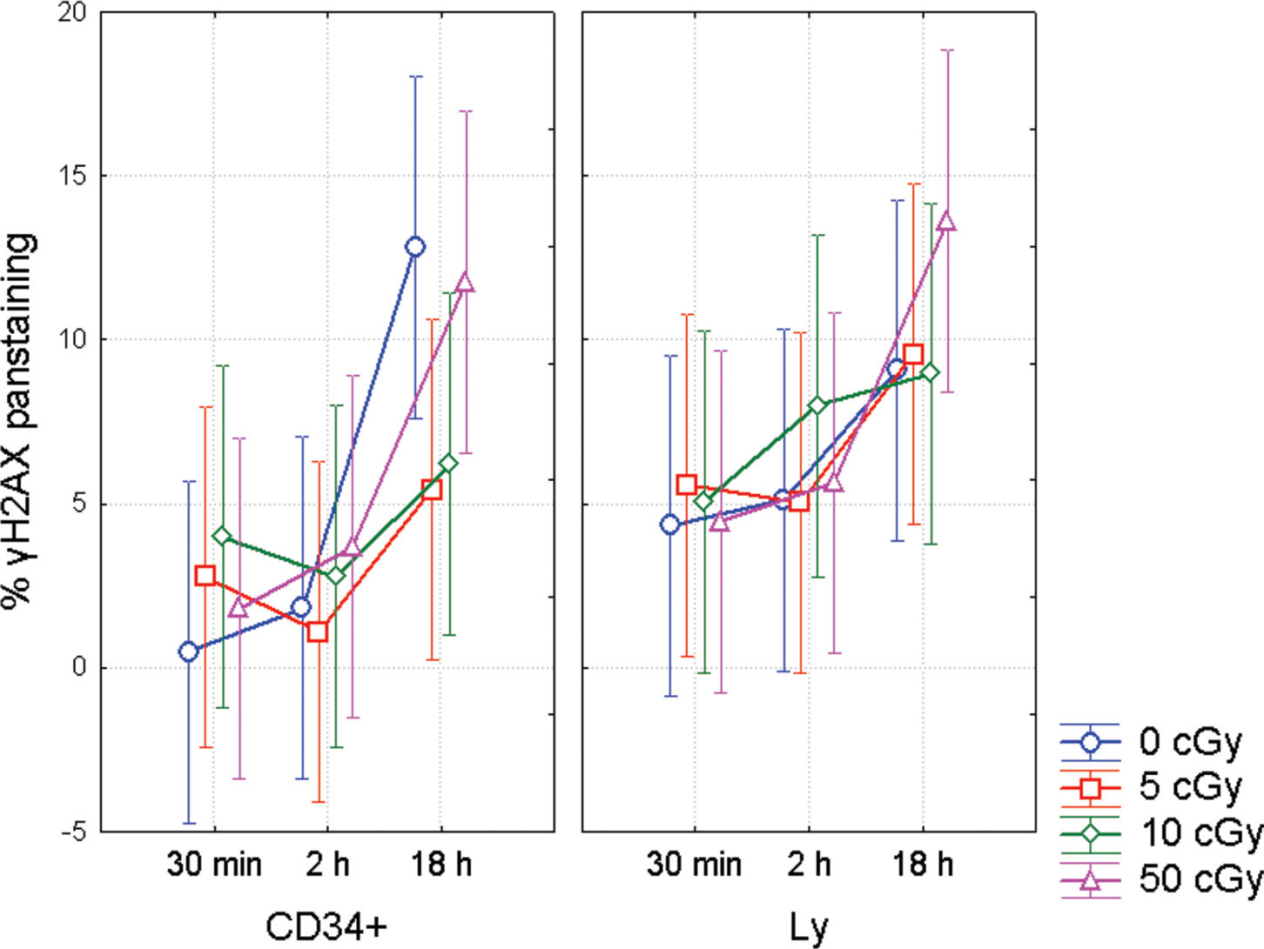
γH2AX pan-staining in CD34+/− UCB cells γH2AX pan-staining in UCB lymphocytes (Ly) and CD34+ HSPC from UCB as analyzed by the imaging flow cytometry. MNC were analyzed in the different time points after irradiation with γ-rays (0, 5, 10, and 50 cGy). In each data point, mean from 4 experiments along with 95% confidence interval is shown.

In line with our previous study [10], we analyzed γH2AX pan-staining cells that are believed to occur in early apoptosis and observed two different types of γH2AX pan-staining. The first one appeared as staining in vicinity of nuclear membranes, so called apoptotic ring, and the second one resulted in homogeneous staining of the whole nucleus (Figure 5). Analysis of all data for γH2AX pan-staining has shown significant difference between the CD34+ and CD34- cells (ANOVA, *p* = 0.02) (Figure 6). Also endogenous level of pan-staining was significantly lower in CD34+ cells after 30 min incubation post sham manipulation (*t-test*, *p* = 0.002). These data confirmed the results of flow cytometry analysis, which showed lower rate of endogenous and radiation-induced apoptosis in CD34+ cells (Figure 2).

## DISCUSSION

In this work we have studied apoptosis and DNA damage response induced by low and therapeutic doses of ionizing radiation in UCB derived CD34+ HSPC and lymphocytes. There was some variation between experiments that could be accounted for interindividual variability of probands. In contrast to other studies, we used frozen UCB cells, what may also contribute to this variability. However, the error bars in our study are comparable with other reports for DNA repair foci [9–11, 15], apoptosis [8], and pan-staining [10].

Until now, there is insufficient experimental evidence considering DNA damage response of HSPC to ionizing radiation, especially in comparison of UCB HSPC with more differentiated cell populations. Kraft et al. analyzed DNA damage response in activated CD34+ cells derived from peripheral blood as compared to peripheral blood lymphocytes [20]. In line with our results, these authors did not observe difference in the γH2AX foci level, but higher level of 53BP1 foci in CD34+ population was found. These data are also in line with the previously published results obtained by our group [10]. Milyavsky et al. compared DNA damage response in different subpopulations of CD34+ cells: termed HSPC (Lin-CD34+CD38-CD45RA-CD90+), Multipotent progenitors (MPP) (Lin-CD34+CD38-CD45RA-CD90-), and progenitors (Lin-CD34+CD38+) [8]. These authors observed higher level of radiation induced γH2AX foci in the merged HSC/MPP population compared to progenitors 12 h after 3 Gy of IR, but they did not compare HSPC with lymphocytes. Vandevoorde et al. enumerated γH2AX/53BP1 foci in UCB derived CD34+ and T-lymphocytes and also adult T-lymphocytes [9]. No difference was observed between populations 30 min after low dose X-ray irradiation. However, higher level of γH2AX/53BP1 foci was found 24 h post-irradiation with 4 Gy of X-ray in CD34+ population compared with T-lymphocytes. Rube et al. analyzed γH2AX foci in CD34+ and CD34- cells in correlation with age of probands [11]. The level of γH2AX endogenous foci observed by these authors in the newborns (0.1 for CD34+ and 0.07 for CD34-) is very similar to our results (0.06 for CD34+ and 0.17 for CD34-) while the level of radiation induced γH2AX foci was slightly lower in our study. Based on the results of our present and previous study [10] we concluded that there is no difference in formation of γH2AX foci between HSPC and lymphocytes. On the other hand, we observed a trend to lower number of endogenous γH2AX foci in CD34+ cells.

In present work, for the first time, we have studied apoptosis induced by ionizing radiation at low doses in HSPC CD34+ cells in comparison to CD34- cells. Our data obtained with standard flow cytometry have shown much lower rate of apoptosis in CD34+ cells. We have also studied very early stage of apoptosis by analyzing γH2AX pan-staining with imaging flow cytometry. Two types of γH2AX pan-staining, complete nuclear staining and so-called apoptotic rings representing ring shaped γH2AX staining on the periphery of the cell, which co-localize with the TUNEL assay staining [21], were analyzed. In line with our Annexin/7-AAD flow cytometry data, CD34- cells have shown significantly higher level of γH2AX pan-staining as compared to CD34+ cells, thus confirming that HSPC are less prone to undergo endogenous and radiation induced apoptosis. So far, very few studies have analyzed apoptosis in hematopoietic stem cells. Milyavsky et al. compared apoptosis in different subpopulations of CD34+ cells, termed HSPC (Lin-CD34+CD38-CD45RA-CD90+), MPP (Lin-CD34+CD38-CD45RA-CD90-), and progenitors (Lin-CD34+CD38+) [8]. Higher percentage of both Annexin and 7AAD positive cells was observed in more primitive CD34+ fractions 18 h after irradiation. However, these authors did not compare bulk CD34+ population with lymphocytes and effects of low dose irradiation was not studied either. Milyavsky et al. also observed higher expression of apoptosis promoting proteins in CD34+CD90+ population compared to CD34+CD90-. On the other hand, Fukuda et al. reported higher expression of protein survivin, that acts as an inhibitor of apoptosis, in the UCB derived CD34+ cells compared to the CD34- population [17]. Merkerova et al. analyzed expression profiles of miRNA clusters in UCB and showed that one of the miRNA clusters, specifically miR-17-92 was highly expressed in the UCB CD34+ cells compare to more differentiated populations [19]. miR-17-92 cluster is associated with low level of TGFBR (transforming growth factor beta receptor) expression and is known to has a role in the inhibition of apoptosis [22]. Moreover, Inoue et al. have shown that transcriptional factor *Slug* is induced by p53 upon irradiation and then protects the damaged cells from apoptosis by directly repressing p53-mediated transcription of *Puma* [23]. This transcriptional factor is preferentially expressed in HSPC, and it shifts the fate of hematopoietic stem and progenitor cells after genotoxic stress toward survival by selectively blocking the apoptotic pathway triggered by p53 [24]. Mohr et al. have shown selective expression of an anti-apoptotic caspase-8L isoform, but not pro-apoptotic caspase-8a/b isoforms, in human CD34+ HSPC [18]. Aforementioned publications [17–19, 23, 24] support our results showing that UCB-derived CD34+ cells are less prone to undergo apoptosis than UCB lymphocytes and accounting this radioresistance for higher expression of anti-apoptotic proteins in the CD34+ cells. Our results are in line with the concept of “apoptosis prone and reluctant” cells, which is useful for practical application by targeting selected cells for example in treatment of cancer with poor curability [25].

## MATERIALS AND METHODS

### Chemicals

Reagent grade chemicals were obtained from Sigma-Aldrich (St. Louis, Missouri, USA) and Life technologies (Carlsbad, California, USA).

### Ethical considerations

This study has been approved by the Ethics Committee of Children's Hospital in Bratislava.

### Cells

Mononuclear cells (MNC) were extracted from UCB as previously described [10] and stored in liquid nitrogen before experiments. In total, UCB cells from 11 different probands were used. Adherent monocytes were removed by incubation of MNC for 2 h in RPMI medium supplemented with 10% FBS and 100 IU/ml penicillin, 100 μg/ml streptomycin, at 37°C in a 5% CO_2_-incubator. Viability of remaining MNC was higher than 95% as defined by the Trypan blue exclusion assay.

### Irradiation

The cells were irradiated on ice by γ-rays at the dose rate of 0.35 Gy/min using a *THERATRON^®^ Elite 80* (MDS Nordion, Ottawa, Canada). The irradiated cells were prewarmed to 37°C in a water bath and then incubated at 37°C in a CO_2_-incubator till analysis. Control cells were concurrently subjected to the same manipulations as irradiated ones.

### Imaging flow cytometry

UCB cells from four different probands were analyzed by imaging flow cytometry for γH2AX foci and pan-staining. After incubation for desired period of time, 3–5 million cells were fixed and immunostained with the primary antibody: γH2AX polyclonal/rabbit at 1:100 dilution (Cell Signalling Technology, Danvers, Massachusetts, USA) as described previously [14]. Afterwards the secondary antibody Alexa Fluor 488 anti-rabbit 1:200 (Life technologies, Invitrogen Molecular probes, New York, USA) and monoclonal anti-human CD34 APC-conjugated antibody 1:15 (Miltenyi Biotec, Bergisch Gladbach, Germany) were added and incubated for 1 hour at room temperature in the dark. Subsequently, the cells were washed in cold PBS and stained with 3 μM DAPI (Life technologies, Invitrogen Molecular probes). From each sample, at least 15000 cells were captured using the ImageStream^X-100^ (Amnis Inc., Seattle, Washington, USA) with 60x objective and the extension depth of field 1 (EDF1) to gain the best possible resolution. Four lasers, 405, 488, 656, 785 nm and CCD camera were used to analyze DNA, γH2AX, CD34, granularity and cell morphology, respectively. Images of cells were acquired at the rate of 50-200 cell/s.

Using the IDEAS software, image compensation was performed and IRIF were enumerated in appropriate cells as previously described [14].

### Flow cytometry

UCB cells from eleven different probands were analyzed for apoptosis by flow cytometry. The cells were harvested in different time points after irradiation (0, 18 and 42 h) spun down (100 g/10 min), washed with PBS and resuspended in 100 μl of the Annexin kit buffer (Roche, Basel, Switzerland). Cells were then stained with Annexin-V (BD biosciences, San Jose, California, USA), 7-AAD (BD biosciences) and the antibodies against the cell surface markers: anti-human CD45-V450 (BD biosciences) and anti-human CD34-APC (Miltenyi Biotec). Samples were then incubated for 20 min in dark, washed with PBS, spun down, diluted in 200 μl of the Annexin kit buffer and analyzed by the BD FACS Canto II flow cytometer (BD biosciences). Compensation was performed on samples where early apoptotic and LAN cells were present in higher percentage. Single color stained tubes and unstained controls were acquired and compensation was generated automatically by the BD FACSDiva software.

### Statistics

Analysis of variance (ANOVA) was carried out using Statistica 8.0 (Dell software, Round Rock, Texas, United States). Comparison between treatment conditions was performed using two tailed *t-test*. The results were considered significantly different at *p* < 0.05

## Abbreviations

DSB: DNA double-strand
breaks *γ*H2AX: phosphorylated histone 2A family member X
HSC: Hematopoietic stem cells; histone 2A family member X
HSPC: Hematopoietic stem/progenitor cells
IRIF: ionizing radiation induced foci
Ly: lymphocytes
LAN: late apoptotic and necrotic
MNC: Mononuclear cells
UCB: Umbilical cord blood
7-AAD: 7-Aminoactinomycin D
